# Alteration of Cellular Energy Metabolism through LPAR2-Axin2 Axis in Gastric Cancer

**DOI:** 10.3390/biom12121805

**Published:** 2022-12-02

**Authors:** Hosne Ara, Utsab Subedi, Papori Sharma, Susmita Bhattarai, Sudha Sharma, Shrivats Manikandan, Xiuping Yu, Md. Shenuarin Bhuiyan, Hong Sun, Sumitra Miriyala, Manikandan Panchatcharam

**Affiliations:** 1 Department of Cellular Biology and Anatomy, Louisiana State University Health Sciences Center, Shreveport, LA 71130-3932, USA; 2 Department of Biochemistry and Molecular Biology, Louisiana State University Health Sciences Center, Shreveport, LA 71130-3932, USA; 3 Department of Pathology and Translational Pathobiology, Louisiana State University Health Sciences Center, Shreveport, LA 71130-3932, USA

**Keywords:** gastric cancer, cell migration, lysophosphatidic acid, β-catenin, Axin2, LPAR2 receptor

## Abstract

Lysophosphatidic acid (LPA), a multifunctional endogenous phospholipid, plays a vital role in cellular homeostasis and the malignant behavior of cancer cells through G-protein-coupled receptors. However, the role of LPA in β-catenin-mediated gastric cancer is unknown. Here, we have noted the high expression of LPAR2 in human gastric cancer tissues, and that LPA treatment significantly increased the proliferation, migration, and invasion of human gastric cancer cells. Results from our biochemical experiments showed that an LPA exposure increased the expression of β-catenin and its nuclear localization, increased the phosphorylation of glycogen synthase kinase 3β (GSK-3β), decreased the expression of Axin2, and increased the expression of the target genes of the β-catenin signaling pathway. The LPA2 receptor (LPAR2) antagonist significantly reduced the LPA-induced nuclear localization of β-catenin, the primary signaling event. The knockdown of LPAR2 in the gastric cancer cell lines robustly reduced the LPA-induced β-catenin activity. An LPA exposure increased the ATP production by both oxidative phosphorylation and glycolysis, and this effect was abrogated with the addition of an LPAR2 antagonist and XAV393, which stabilizes the Axin and inhibits the β-catenin signaling pathway. Based on our findings, the possibility that LPA contributes to gastric cancer initiation and progression through the β-catenin signaling pathway as well as by the dysregulation of the energy metabolism via the LPAR2 receptor and Axin2, respectively, provides a novel insight into the mechanism of and possible therapeutic targets of gastric cancer.

## 1. Introduction

Despite recent advances in diagnosing and treating gastric cancer, it remains the third most common cause of cancer-related death globally, especially in Asia, Europe, and the United States [1]. Distinct phenotypes of gastric cancer occur due to specific genetic alterations and epigenetic changes. Although different groups have reported the dysregulation of many signaling pathways, proteins, and multiple genes in gastric cancer, its mechanisms of carcinogenesis and heterogeneity remain unclear [2,3]. About 70% of gastric cancer patients show the dysregulation of the three major signaling pathways, including the Wnt β-catenin pathway, the nuclear factor κB (NFκB) pathway, and proliferation/stem cell pathways. Among these, dysregulated β-catenin pathways have been reported in 30–50% of gastric cancer patients and in different gastric cancer cell lines [4,5]. In addition, other types of human cancers are initiated by the mutation of other components of the β-catenin signaling pathway, such as β-catenin, adenomatous polyposis coli (APC), and Axin [6,7].

Several studies have demonstrated that β-catenin signaling is activated by the mutation of these components and a variety of extracellular stimuli [7,8]. For example, Wnt proteins are well-known proteins that bind with the Frizzled receptors and activate the β-catenin pathway [9]. In addition, recent studies have revealed that β-catenin signaling is activated by GPCR signaling, and LPA acts through its G-protein-coupled receptor [8].

Lysophosphatidic acid (LPA) is a multifunctional endogenous phospholipid that mediates the proliferation, differentiation, migration, regulation of cell–cell interaction [10], inhibition of cell death [11], neurite retraction [12], smooth muscle cell contraction [13], and transformation and progression in cancer [14]. LPA binds mainly with its six specific G-protein-coupled receptors (GPCR) to mediate the cellular responses. The aberrant expression of LPA receptors, particularly LPAR2 and LPAR3, has been reported in several cancers, suggesting a significant role for LPA in different cancer types through distinct signaling pathways such as AKT, MAPK, and ERK [15]. In this study, we show for the first time that LPA stimulates the proliferation, migration, and invasion of gastric cancer through the β-catenin signaling pathway and by increasing the energy metabolism, providing a novel insight into the molecular mechanism of gastric cancer.

## 2. Materials and Methods

### 2.1. Materials

LPA was purchased from Avanti Polar Lipids (Birmingham, AL, USA). Ki16425, an LPA antagonist against LPAR1, LPAR2, and LPAR3, was purchased from Echelon (Salt Lake City, UT, USA), and the LPAR2 antagonist (Cat # HY-18075) was purchased from MedChem Express (Monmouth Junction, NJ, USA) Rabbit monoclonal anti-β-catenin and rabbit polyclonal anti-LPA1# ab23698, LPA2#ab38322, LPA3#NBP1-84903, and LPP3 were purchased from Abcam (Cambridge, MA, USA). Antibodies against β-actin#3700 and GAPDH#97166 were purchased from Cell Signaling Technology (Beverly, MA, USA). Antibodies against c-Myc#13987T were purchased from Santa Cruz Biotechnology, Inc. (Santa Cruz, CA, USA). Tissue lysates for the total protein were purchased from Novus Biologicals, LLC (Cat #NBP2-47080, NBP2 47081, and NBP2 47082). cDNA (Cat #C1234248, C1235248), RNA (Cat #R8235248-PP-10, R1235248-50, R1234253-10), and paraffin-embedded human tissue sections from the normal stomach and cancerous stomach (Cat# T8235248-PP) were purchased from Biochain (Newark, CA, USA). The LPA/Lysophosphatidic Acid (Competitive EIA) ELISA Kit-LS-F25111 was purchased from LSBio (Seattle, WA, USA).

### 2.2. Cell Culture

We purchased the AGS #CRL-1739 and NCI-N87 (ATCC Cat# CRL-5822, RRID: CVCL_1603) human gastric cancer cell lines from American Type Culture Collection (ATCC; Rockville, MD, USA). An ATCC-formulated F12 K medium was used for the AGS cell line and an RPMI-1640 medium was used for the NCI- N87 cell line. Both cell lines were supplemented with 10% fetal bovine serum (FBS) (Sigma, St. Louis, MO, USA), 100 U/mL penicillin, and 100 µg/mL streptomycin (Corning, NY, USA), maintained at a temperature of 37 °C and at 5% CO_2_ and sub-cultured by trypsinizing with trypsin-EDTA solution (Gibco, Gaithersburg, MD, USA).

### 2.3. LPA Treatment

The AGS and NCI-N87 cell lines were maintained throughout the study in the presence of 10% FBS F12K and 10% FBS RPMI medium, respectively. LPA (10 µM) was prepared with 1% (*w*/*v*) fatty acid-free BSA; before any treatment with LPA, the cells were starved in the presence of 0.1% dilapidated FBS F12 K or an RPMI medium for 24 h.

### 2.4. Cell Proliferation Assay

The cell proliferation assay was performed using the electric cell-substrate impedance sensing (ECIS) method, which measures the cell proliferation activity in real-time and by a hemocytometer. For the measurement using the hemocytometer, 0.5 × 105 cells were seeded in 6-well plates containing a complete growth medium and allowed to grow for 24 h. The cells were then treated with 10 µM of LPA after 24 h of starvation as described previously. The total number of cells in each well was counted every 24 h for 3 days after an LPA treatment. For the measurement using the ECIS proliferation assay, 15,000 cells were suspended in a complete growth medium were cultured on the surface of gold-film electrode-coated 8W20idf PET 8-well plates, which have ten small electrodes connected in parallel in each well. Once the cells were attached to the bottom, we treated the cells with the control (no LPA), 10 µM of LPA, an LPA + LPAR2 antagonist, or LPA+ Ki16425. The impedance was measured every 10 s at a frequency of 4000 for 60 h.

### 2.5. ECIS Wound-Healing Assay (ECIS)

An ECIS wound healing assay was performed using ECIS-Applied Biosystem technology. For the ECIS wound healing assay, 50,000 cells were seeded into the ECIS well and allowed to grow until a confluent monolayer formed and the cells entered the stationary phase. The cells were starved for 24 h after entering the stationary phase. After 24 h of starvation, electrical wounds were made in the ECIS plate by elevating the voltage pulse of a 40 kHz frequency at 3.5-V amplitude for 30 s. This sudden elevation in the voltage causes cell death and detachment from the active electrode. The medium was then aspirated and treated with 10 µM of LPA and the wound healing was assessed using continuous statistical analysis. The effect of Ki16425 and LPAR2 antagonists on the migration activity was measured using cells pretreated with 5 µM of LPAR2 antagonist or 10 µM of Ki16425 for 1 h and then exposed to 10 µM of the LPA +LPAR2 receptor antagonist or Ki16425.

### 2.6. Luciferase Reporter Assay

We transfected the AGS or NCI-N87 cells with the TOP flash luciferase reporter gene plasmid using the X-treme gene transfection reagent #0636624001(Rockville, MD, USA) according to the manufacturer’s instructions. We changed the medium of the transfected cells to a starvation medium after 24 h of transfection and maintained the cells under starvation conditions for 8 h. The serum-starved cells were treated with or without 10 µM of LPA for 24 h. For the treatment with inhibitors, serum-starved cells were pretreated with 10 µM of Ki16425 or 5 µM of LPAR2 antagonist for 1 h, followed by a co-treatment with 10 µM of LPA along with the respective inhibitors. Luminescence was measured in a single tube luminometer using the Promega luciferase assay system (E1500; Madison, WI, USA) and was normalized by the total protein concentration.

### 2.7. Mitochondrial Bioenergetics

We analyzed the OCR (oxygen consumption rate) and ECAR (extracellular acidification rate) using a Seahorse XF-24 extracellular flux analyzer (Seahorse Biosciences, Chicopee, MA, USA). Gastric cancer cells were grown in a Seahorse plate until they became fully confluent and treated with the control (no LPA), LPA (10 µM), or LPA plus the inhibitors. The bioenergetic activity of cells with or without an LPA treatment was monitored as free protons in real-time using the Seahorse XF-24 analyzer to measure the oxygen concentration. We quantified the OCR and ECAR values in pmol/min/μg and mpH/min/μg, respectively, and normalized the values to the total protein concentration. The initial basal value of the OCR was interrupted by the addition of oligomycin (Complex V inhibitor), giving values for the ATP-linked OCR. The addition of FCCP (an uncoupler) and rotenone + antimycin (Complex I and Complex III inhibitor) allowed us to determine the maximal OCR capacity and spare OCR capacity, respectively. We used a glucose-free medium for the ECAR analysis. Following the sequential addition of glucose (25 mM), oligomycin (1 μg/mL), and deoxyglucose (25 mM), we measured the rate of glycolysis and glycolytic reserve in the gastric cancer cells.

### 2.8. Data Analysis from the Clinical Cohort

We used a gastric cancer TCGA data set obtained from the Cancer Genome Atlas (TCGA) through the UALCAN (UALCAN, RRID: SCR_015827; http://ualcan.path.uab.edu/cgi-bin/ualcan-res.pl; accessed on 1 November 2018) webserver to analyze the mRNA expression level of different LPA receptors and β-catenin mRNA levels [16].

### 2.9. Statistical Analysis

Unless otherwise stated, the data are reported as means ± (SD). We repeated our in vitro experiments a minimum of three times. We used a one-way ANOVA with a Bonferroni correction for a significant correlation among the different groups. In addition, an unpaired Student’s *t*-test was used to identify the significant differences between the two groups. A statistical analysis was performed using prism 8.0 (GraphPad Prism, version 8.4.2, San Diego, CA, USA, RRID: SCR_002798).

## 3. Results

### 3.1. Upregulation of LPA and LPAR2 in Gastric Cancer

**Figure 1:**
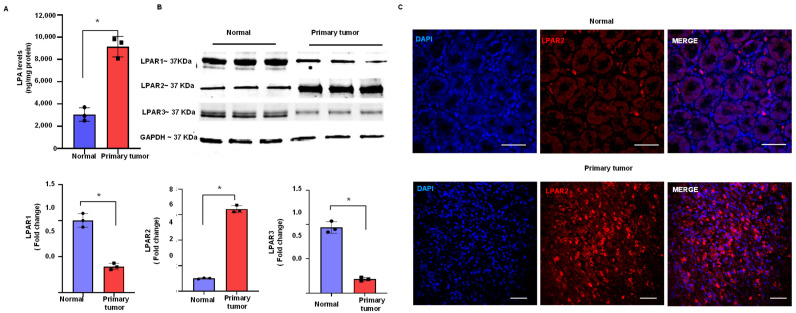
Upregulation of LPA and the LPAR2 receptor in human stomach cancer. (**A**) LPA ELISA immunoassay was performed in samples of the normal stomach (Normal) and stomach cancer (Primary tumor) tissue. (**B**) Upper panel: immunoblot analysis of LPAR1-3 receptors in normal stomach and primary tumor tissue. We used GAPDH as a loading control. Lower panel: quantification of the Western blotting result. (**C**) Immunohistochemical analysis of LPAR2 in normal stomach and stomach cancer tissue sections (scale bar: 50 µm). All values are mean ± SD (*n* = 3). * *p* < 0.05 compared to normal stomach tissue using Student’s *t*-test.

First, to evaluate the relevance of LPA in human gastric cancer, we measured the LPA levels in the normal human stomach and gastric cancer tissues. We found increased LPA levels (*p* < 0.001) in gastric cancer tissue compared to those in the normal stomach (Figure 1A). Next, we analyzed the mRNA levels of different LPA receptors in a clinical cohort of gastric cancer using interactive web resources (UALCAN) [16]. Our TCGA dataset analysis suggested that different LPA receptors are expressed to a different extent in human gastric cancer tissues (*n* = 415) compared to the expression in the normal stomach tissues (*n* = 34); in particular, the levels of LPAR2 were greatly increased (*p* < 0.05) in human gastric cancer tissues (Supplementary Figure S1A). In addition, the LPAR2 mRNA level was increased in all stages of gastric cancer (Supplementary Figure S1B). Subsequently, we performed the Western blotting analysis of LPAR1-3 receptors and we found that the LPAR2 protein expression was significantly increased (*p* < 0.001) in the human stomach tumor compared to the normal human stomach tissue (Figure 1B). In addition, the immunohistochemical analysis of LPAR2 in the normal human stomach and primary tumor of the stomach tissue sections showed a significant increase in the LPAR2 expression in gastric cancer tissues (Figure 1C).

Based on our literature review of gastric cancer [17,18,19] and the preliminary data obtained from human gastric cancer tissues [20], we investigated the LPA-LPAR2-mediated β-catenin signaling pathway in gastric cancer. Our study used two gastric cancer cell lines, the AGS and NCI-N87 cell lines.

Next, to confirm the LPAR2 expression in our AGS and NCI-N87 cell lines we performed RT-PCR and Western blotting analysis and found that the LPAR2 expression was remarkably increased in both cell lines compared to the GES normal gastric epithelial cell line (Supplementary Figure S1C,D). These results suggest that LPA plays a definitive role in gastric cancer through the LPAR2 receptor.

### 3.2. LPA Stimulates Proliferation of Gastric Cancer Cells through LPAR2

**Figure 2:**
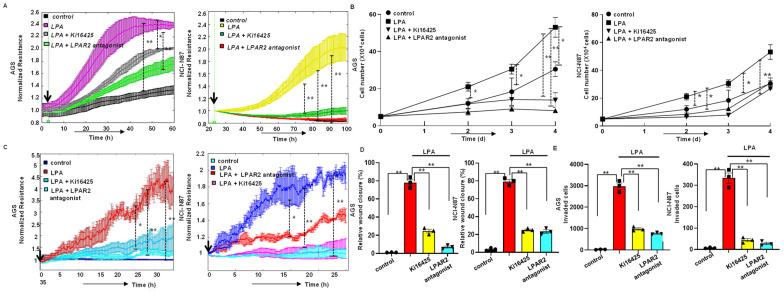
LPA increased the proliferation of the gastric cancer cell line through the LPAR2 receptor. (**A**) Effect of the LPAR-1/ LPAR2 and LPAR-3 inhibitor Ki16425 and LPAR2 antagonist on LPA-stimulated ECIS proliferation activity in the AGS and NCI-N87 cell line. Left panel: AGS cell line (*n* = 3): blackline, control; purple line, LPA (10 µM); grey line, LPA+ Ki16425; green line, LPA+ LPAR2 antagonist. Right panel: NCI-N87 cell line (*n* = 3): black line, control; yellow line, LPA (10 µM); green line, LPA+ Ki16425; red line, LPA+ LPAR2 antagonist. (**B**) Hemocytometer proliferation assay showing that both the LPAR2 antagonist and Ki16425 significantly reduced LPA-induced proliferation activity in the AGS cells (left panel) or NCI-N87 cells (right panel). All values are mean ± SD (*n* = 3). * *p* < 0.05, ** *p* < 0.01; one-way analysis of variance (ANOVA) followed by the Bonferroni post hoc test. (**C**–**E**) Effect of the LPA receptor antagonists on LPA-induced migration and invasion of the gastric cancer cell line. (**C**) Left panel: ECIS wound healing assay analysis of the effect of Ki16425 on LPA-induced migration activity in the AGS cell line. Dark blue line, control; red line, LPA (10 µM); dark turquoise blue line, LPA+ Ki16425; and light turquoise blue line, LPA+ LPAR2 antagonist. Right panel: ECIS wound healing assay in NCI-N87 cell. Light turquoise blue line, control; dark blue line, LPA (10 µM); red line, LPA+ Ki16425; purple line, LPA+ LPAR2 antagonist. (**D**) Scratch assay analysis of the effect of Ki16425 and LPAR2 antagonist on LPA-induced migration in the gastric cancer cells. Quantified wound closure rate after 24 h of exposure to LPA in the presence and absence of LPA inhibitors in AGS (left) or NCI-N87 (right) cells. (**C**). LPA receptor antagonists reduced LPA-induced invasive activity in gastric cancer cells. Quantification of the results of the invasion assay. Left panel: AGS cell line; right panel: NCI-N87 cell line. All values are mean ± SD (*n* = 3). ** *p* < 0.01; one-way ANOVA) followed by the Bonferroni post hoc test.

To evaluate the functional role of the LPA in the gastric cancer cell lines, we performed ECIS proliferation assays to measure the proliferation of gastric cancer cells in real time. An increased resistance was observed as the number of cells on the gold-plated electrode increased. We also confirmed this result by a hemocytometer. First, we performed real-time ECIS proliferation assays by treating the AGS cells with 5, 10, or 20 µM of LPA to determine the optimal LPA concentration for our experiments. We found that the LPA-induced proliferation of the AGS cell line in a dose-dependent manner (Supplementary Figure S2A). Since LPA (10–20 µM) caused a robust increase in the proliferation of gastric cancer cells, we selected 10 µM of LPA as the optimal dose for the rest of our experiments. We also observed that the LPA treatment significantly increased (*p* < 0.01) the number of cells compared to the control group not exposed to LPA in both the ECIS proliferation assay and using a hemocytometer (Figure 2A,B and Supplementary Figure S2B). Next, we investigated the effect of a specific LPAR2 antagonist as well as Ki16425, which is an LPAR_1–3_ receptor antagonist, on the LPA-induced proliferation activity. The results from our ECIS and hemocytometer proliferation assays showed that the LPA-induced proliferation activity of the gastric cancer cells was significantly abrogated (*p* < 0.01) by the LPAR2 antagonist as well as by the treatment with Ki16425 (Figure 2A,B and Supplementary Figure S2B), and that the effect of the LPAR2 antagonist was similar to that of Ki16425.

### 3.3. LPA-Induced Motility of Gastric Cancer Cells through LPAR2 Receptor

Next, we examined the effect of the LPA on the gastric cancer cell migration activity using an ECIS migration assay and a scratch assay. This showed that an LPA treatment significantly increased (*p* < 0.01) the migration activity of the gastric cancer cells in both the ECIS migration assay and the scratch assay. This LPA-induced migration activity was abrogated with the addition of Ki1645 as well as the LPAR2 antagonist (Figure 2C,D and Supplementary Figure S2C,D). Subsequently, to assess whether motile ability accompanied the invasive activity, we performed a transwell invasion assay in the presence or absence of an LPA treatment. Remarkably, the invasion activity was drastically increased (*p* < 0.01) after the LPA treatment, while the treatment with Ki16425 or the LPAR2 antagonist significantly reduced (*p* < 0.01) the LPA-induced invasion activity (Figure 2E and Supplementary Figure S2E).

### 3.4. LPA Mediates Gastric Cancer through LPAR2 and the β-Catenin Signaling Pathway

To understand the molecular mechanism responsible for LPA-mediated gastric cancer, we measured the mRNA levels of β-catenin in normal healthy gastric epithelial cells and in the AGS cell line. Our RT-PCR results revealed the significant upregulation of β-catenin in the AGS cell line compared to normal healthy cells (Supplementary Figure S1E). Again, when we treated the AGS cell line with LPA; to our surprise, the LPA treatment significantly increased the β-catenin expression in a time-dependent manner (Supplementary Figure S1F).

Subsequently, we found that LPA significantly increased the phosphorylation of GSK-3β at ~4 h and persisted to 8 h following the LPA treatment in both cell lines (*p* < 0.05, *p* < 0.01) (Supplementary Figure S4B). To investigate Axin2, which has been reported to be a negative regulator of the β-catenin signaling pathway, we performed an RT-PCR of Axin2 in both cell lines after an LPA treatment. Our RT-PCR results showed that the mRNA level of Axin2 was decreased significantly (*p* < 0.01) in LPA-treated cells compared to that in the control cells (Supplementary Figure S4A). These results indicate that an LPA treatment reduces the GSK-3β and Axin2 levels, thereby disrupting the β-catenin destruction complex and ultimately leading to the β-catenin pathway’s activation.

**Figure 3:**
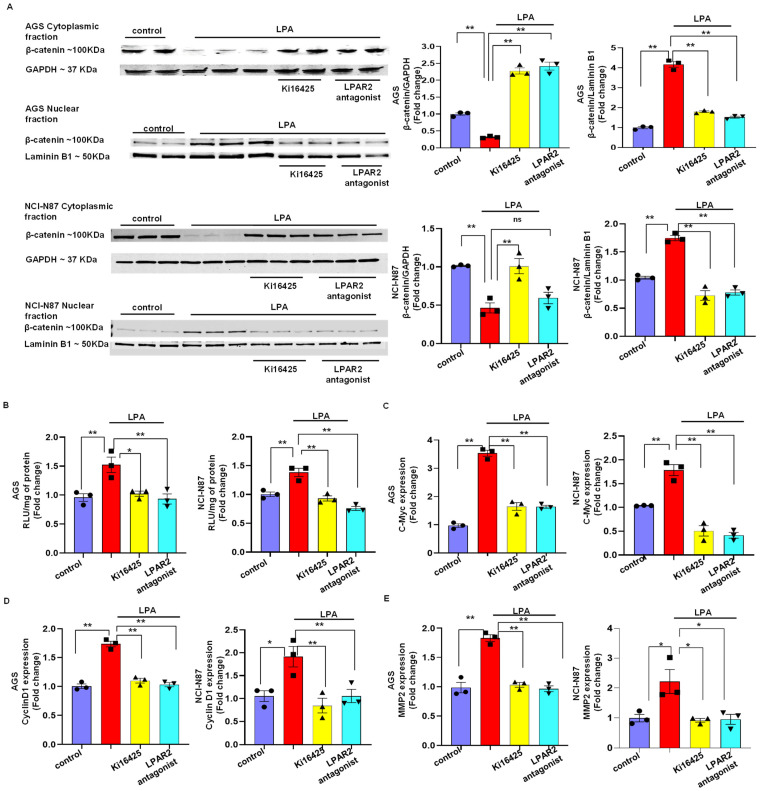
LPA treatment activated the β-catenin signaling pathway through the LPAR2 receptor. (**A**) The cytoplasmic and nuclear fraction of AGS (**upper**) and NCI-N87 cells (**lower**) were loaded on an SDS-PAGE gel and β-catenin was detected using Western blotting. We used GAPDH and laminin B1 as fractionation and loading controls. (**B**) Luciferase assay examining the effect of LPA, Ki16425 or LPAR2 antagonist in AGS (**left**) and NCI-N87 (**right**) cells. RLU, relative light unit. (**C**) Effect of LPA receptor antagonists on LPA-induced increased expression in the target genes of the β-catenin signaling pathway in AGS (**left panel** of **C**–**E**) and NCI-N87 (**right panel** of **C**–**E**) cell line. All values are mean ± SD (*n* = 3). * *p* < 0.05, ** *p* < 0.01 compared to the control. Student’s *t*-test was used for comparisons between two groups; one-way ANOVA followed by the Bonferroni post hoc test was performed for more than two groups.

To examine whether LPA activates the β-catenin signaling pathway through LPAR2, we measured the effect of the LPAR2 antagonist and Ki16425 on the nuclear localization of β-catenin in both cell lines using Western blotting analysis and a luciferase assay. The Western blotting analysis showed that an LPA treatment significantly increased (*p* < 0.01) the nuclear fraction of β-catenin. In contrast, the LPA-induced nuclear fraction of β-catenin was significantly reduced (*p* < 0.01) when the cells were treated with the LPAR2 antagonist or Ki16425 (Figure 3A). Finally, to delineate the role of the LPA2 receptor on the LPA-induced T-cell factor/lymphoid enhancer-binding factor (TCF/LEF)/β-catenin transcriptional activity, we performed a luciferase assay. The results from our luciferase assay showed that an LPA treatment significantly increased (*p* < 0.01) the β-catenin transcriptional activity, and the LPAR2 receptor antagonists significantly abrogated (*p* < 0.01) the β-catenin transcriptional activity in both the NCI-N87 cell line and the AGS cell line (Figure 3B).

Next, we examined the mRNA levels of c-Myc and Cyclin D1, which are the critical target genes for the active β-catenin signaling pathway in both cell lines, with or without an LPA treatment [21]. An LPA treatment caused a significant induction of the c-Myc and Cyclin D1 mRNA levels compared to induction in the untreated cells. When the cells were treated with LPA receptor antagonists, both the LPAR2 antagonist and Ki16425 reduced the LPA-induced expression of c-Myc and CyclinD1 in the AGS cell line and the NCI-N87 cell line (Figure 3C,D). Since we observed a significant induction of the transwell invasion after the LPA treatment, we measured the mRNA level of MMP2, which is also a target gene of the β-catenin signaling pathway and degrades the basement membrane. Our RT PCR results showed that the LPA treatment significantly increased the level of MMP2 mRNA compared to that in the control cells and that the treatment with the LPAR2 antagonist or Ki16425 significantly reduced the LPA-induced expression of MMP2 in both cell lines (Figure 3E).

In addition, we investigated a clinical cohort of gastric cancer for the mRNA levels of β-catenin and its downstream target genes in a normal human stomach and a stomach with cancer using the web resource UALCAN. Upon the analysis of the TCGA dataset of 415 human gastric cancer tissue samples and 34 normal stomach samples, a dramatic increase in the mRNA levels of β-catenin (CTNNB1) and its downstream target genes, such as cMyc, CyclinD1 (CCND1), and MMP2, was observed in human gastric cancer tissue samples (Supplementary Figure S7). These results are consistent with our findings and suggest that an increased LPA in gastric cancer could modulate the aggressiveness of gastric cancer through the β-catenin signaling pathway.

### 3.5. Silencing of LPAR2 by shRNA Inhibits LPA-Induced Progression of Gastric Cancer through the β-Catenin Signaling Pathway

**Figure 4:**
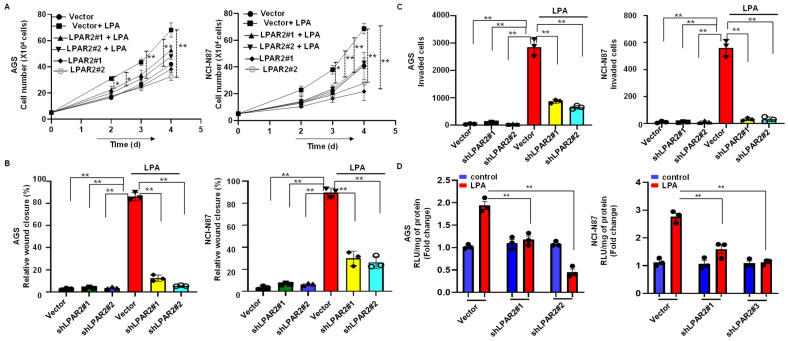
LPAR2 knockdown reduced LPA-induced proliferation, migration, invasion activity, and activation of the β-catenin signaling pathway in the gastric cancer cell. (**A**) AGS (**left**) or NCI-N87 (**right**) cells were lentivirally transduced with vector or three LPAR2-shRNA and subsequently investigated for LPA-induced proliferation using a hemocytometer. (**B**,**C**) LPA-induced migration and invasive activity was reduced in LPAR2 knockdown AGS (**left**) or NCI-N87 (**right**) cells. (**D**) LPAR2 knockdown reduced LPA-induced β-catenin activity in the AGS (**left**) and NCI-N87 (**right**) cell lines. All values are mean ± SD (*n* = 3). * *p* < 0.05, ** *p* < 0.01 compared to the control. Student’s *t*-test was used for comparisons between the two groups; one-way ANOVA followed by the Bonferroni post hoc test was performed for more than two groups.

To confirm that the LPA was mediating its action through the LPAR2 receptor, we generated LPAR2-depleted stable AGS and NCI-N87 cell lines via the lentivirus-mediated transduction of the control and three different LPAR2-shRNA. The knockdown efficiency was variable in the different shRNA transduced cells (Supplementary Figure S5A). When we performed the proliferation assay in LPAR2 knockdown cells by treating the cells with LPA, the LPA-induced proliferation activity was significantly abolished in all LPAR2 knockdown AGS or NCI-N87 cells (Figure 4A). Again, we performed the migration assay in LPAR2 knockdown cells and observed that LPAR2 knockdown significantly reduced the LPA-induced migration activity of both cell lines (Figure 4B and Supplementary Figure S3A). We also confirmed that all of the LPAR2 knockdown cells showed reduced invasive activity even after the LPA treatment in comparison to an LPA-treated empty vector (Figure 4C and Supplementary Figure S3B).

Finally, we examined the effect of an LPAR2 knockdown in the LPA-induced β-catenin activity. We performed the luciferase assay in the LPAR2 knockdown cells and found that an LPAR2 knockdown significantly reduced the LPA-induced β-catenin activity (*p* < 0.01) in all LPAR2-shRNA transduced AGS and NCI-N87 cell lines. Interestingly, the most significant reduction was observed in the LPAR2 shRNA#2 knockdown AGS cells and shRNA#1 and shRNA#3 knockdown NCI-N87 cells, which is consistent with our Western blotting results, shown in Supplementary Figure S5A, and reflects the specificity of the experiment (Figure 4D).

### 3.6. LPA Treatment Stimulates Cellular Energy Metabolism through the LPAR2 Antagonist

**Figure 5:**
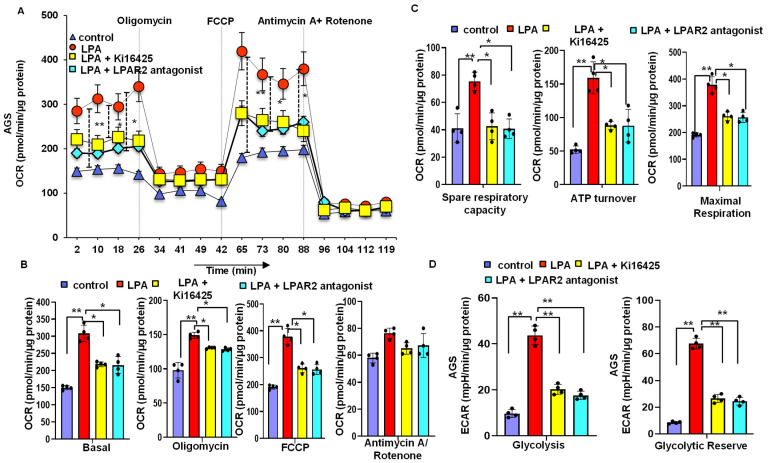
LPA treatment stimulates cellular energy metabolism through the LPAR2 receptor. (**A**,**B**) Representative graphs and quantification of OCR measurement in control (no LPA), LPA-treated, LPA + Ki16425, or LPA + LPAR2 antagonist-treated AGS cells showing basal OCR and after sequential addition of oligomycin, FCCP, and antimycin + rotenone. (**C**) Quantification of spare respiratory capacity, ATP turnover, and maximal respiration after treating the AGS cells with control (no LPA), 10 µM LPA, LPA + Ki16425, or LPA+ LPAR2 antagonist. (**D**) ECAR analysis of the AGS cell line after treating the AGS cells with control (no LPA), LPA, LPA + Ki16425, or LPA+ LPAR2 antagonist. The bar graphs represent glycolysis and glycolytic reserve. All values are mean ± SD (*n* = 5). * *p* < 0.05, ** *p* < 0.01 compared to the control. Student’s *t*-test was used for comparisons between two groups; one-way ANOVA followed by the Bonferroni post hoc test was performed for more than two groups.

When we investigated the mitochondrial bioenergetics of both cell lines using a Seahorse XF24 analyzer [22,23], LPA-treated cells showed an increased basal oxygen consumption rate (OCR) level compared to that in the control cells; with an oligomycin treatment, the OCR level decreased drastically compared to that in the control cells. Additionally, an LPA treatment significantly increased the spare respiratory capacity, ATP turnover, and maximal respiration compared to those in the control cells. In contrast, the LPAR2 antagonist and Ki16425 significantly reduced the LPA-induced basal OCR, spare respiratory capacity, ATP turnover, and maximal respiration (Figure 5A–C). Following extracellular acidification rate (ECAR) analysis, we observed that LPA significantly increased glycolysis and the glycolytic reserve. However, this LPA-induced effect was abolished by a treatment with LPA receptor antagonists (Figure 5D). These results provide strong evidence that an LPA treatment altered the mitochondrial bioenergetics status through the LPAR2 receptor and increased the energy source available to the AGS and NCI-N87 cells by using both mitochondrial OXPHOS and glycolysis.

### 3.7. Stabilization of Axin2 Rescued LPA-Induced Cellular Energy Metabolism and Progression of Gastric Cancer Cells

**Figure 6:**
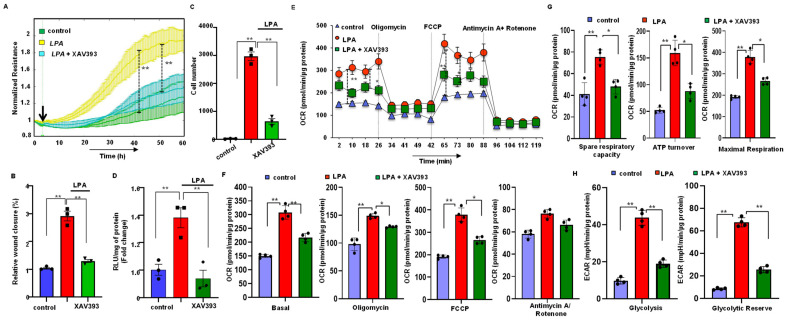
Axin stabilization attenuated LPA-induced progression, β-catenin activation, and mitochondrial energy metabolism in gastric cancer cells. (**A**) Effect of XAV393 on LPA-stimulated ECIS proliferation activity in the AGS cell line (*n* = 3). Green line, control; yellow line, 10 µM LPA; turquoise blue line, LPA+ XAV393. (**B**) Scratch assay analysis of the antagonist effect of XAV393 on LPA-induced migration in gastric cancer cells. Quantified wound closure rate after 24 h of exposure to LPA in the presence and absence of XAV393 in AGS cells. (**C**) XAV393 reduced LPA-induced invasive activity in gastric cancer cells. Quantification of the results of the invasion assay. (**D**) Luciferase assay examining the effect of XAV393 in AGS cells. RLU, relative light unit. (**E**,**F**) Representative graphs and quantification of OCR measurement in control (no LPA), LPA-treated, LPA + XAV393-treated AGS cells showing basal OCR and after sequential addition of oligomycin, FCCP, and antimycin + rotenone. (**G**) Quantification of spare respiratory capacity, ATP turnover, and maximal respiration after treating the AGS cells with control (no LPA), 10 µM LPA, LPA +XAV393. (**H**) ECAR analysis of the AGS cell line after treating the AGS cells with control (no LPA), LPA, LPA + XAV393. The bar graphs represent glycolysis and glycolytic reserve. All values are mean ± SD (*n* = 3). * *p* < 0.05, ** *p* < 0.01; one-way ANOVA) followed by the Bonferroni post hoc test.

We performed an ECIS proliferation assay, wound healing assay, and invasion assay by treating the cells with LPA or LPA with XAV393. Our results indicate that an XAV393 treatment significantly reduced the LPA-induced proliferation, migration, and invasion activity in AGS (Figure 6A–D and Supplementary Figure S6A–C) as well as the NCI-N87 cell line. In addition, when we analyzed the OCR and ECAR in both cell lines after treating the cells with LPA or LPA with XAV393, XAV 393 significantly reduced the LPA-induced OCR and ECAR in AGS (Figure 6E–H) and NCI-N87 cells.

## 4. Discussion

In this report, we have demonstrated for the first time that LPA mediates the initiation and progression of gastric cancer through the LPAR2-β-catenin axis and by altering the energy metabolism. Existing studies in the literature suggest that a high LPA level plays a vital role in various kinds of tumor progression, such as ovarian carcinoma, thyroid carcinoma, pancreatic carcinoma, and colon cancer [24,25,26]. In addition, it has been shown that LPA mediates its downstream signaling pathway through GPCR and modulates various oncogenic signaling [27]. In agreement with this, the results from our clinical cohort studies revealed the high expression of LPAR2 in all stages of gastric cancer tissues compared to the expression in normal gastric tissues. These results suggest a possible role for LPA in the initiation and progression of gastric cancer through the LPAR2 receptor [17]. Data from another study have shown that LPAR-1, LPAR-2, and LPAR-3 are present in gastric cancer cell lines; among them, LPAR2 was expressed aberrantly in the AGS cell line [28]. However, the LPA-LPAR2-mediated functional effect and its downstream molecular signaling mechanism in gastric cancer remain to be elucidated. In this study, we demonstrated that LPA increased the proliferation and subsequent progression of the gastric cancer cells, which get abrogated into the functional level by both LPA receptor antagonists (Ki16425 and the LPAR2 receptor antagonist) and LPAR2 knockdown. Ki16425 is an antagonist of LPA1 and LPA3 with a moderate activity against LPA2 and the LPAR2 antagonist is specific to LPAR2. Since we observed a similar functional effect in both the LPA receptor antagonists and our cell line has a high expression of LPAR2, we speculated that LPA mediates its action through the LPAR2 receptor, which was confirmed by the LPAR2 knockdown.

One of the most common causes of gastric carcinogenesis is the dysregulation of the β-catenin signaling pathway. β-Catenin is a multifunctional protein that maintains cell–cell adhesion by binding with E-cadherin and acts as a transcriptional regulator of β-catenin signaling. When the pathway is inactive, β-catenin binds with the destruction complex composed of Axin, APC, GSK-3β, and casein kinase 1α (CK1α). The phosphorylation of the destruction complex by the kinases leads to the degradation of cytoplasmic β-catenin, resulting in reduced β-catenin levels. In contrast, when the signaling pathway is active, it prevents the phosphorylation of β-catenin, leading to the subsequent stabilization of cytoplasmic β-catenin. A larger amount of cytoplasmic β-catenin then translocates into the nucleus and acts as a transcriptional stimulator of the target genes, such as Cyclin D1, c-MYC, and MMP2, by interacting with the TCF/LEF complex [29,30,31]. According to our findings, LPA induces the initiation and progression of gastric cancer by activating the β-catenin signaling pathway. On the other hand, the LPA-induced β-catenin activity was abrogated with LPA receptor antagonists. Furthermore, an LPA-induced β-catenin activation was confirmed by the knockdown of LPAR2 using LPAR2-shRNAs, which decreased the LPA-induced β-catenin activity.

Since our findings revealed a functional role for LPA in gastric cancer through the activation of β-catenin and its downstream target genes, LPA may provide the oncogenic stimulation that increases the extent of a β-catenin activation and promotes the initiation and progression of gastric cancer. β-catenin cytoplasmic stabilization is due to the disassembly or phosphorylation of any of the β-catenin pathway components, such as APC, β-catenin, Axin, or GSK-3β. It has been shown that the mutation of exon 3 of β-catenin is the most common oncogenic step in human carcinogenesis, including gastric cancer [4]. However, Sekine et al. and Chan et al. showed that when only this deletion mutation occurs, it is not enough to induce carcinogenesis in the HCT116 colon cancer cell line [32,33]. LPA has been reported to phosphorylate GSK-3β in HEK 293 cells [34], and other groups have shown that the phosphorylation of only GSK-3β cannot sufficiently activate the β-catenin signaling pathway [35]. The exogenous overexpression of GSK-3β could not reduce the β-catenin-induced transcriptional activity in β-catenin overexpressing cells, whereas the β-catenin transcriptional activity was reduced after the addition of Axin or APC [36]. In this study, we have shown for the first time that LPA activates the β-catenin signaling pathway by increasing the phosphorylation of GSK-3β and by decreasing the mRNA level of Axin 2, which acts as a negative regulator of the β-catenin pathway (Supplementary Figure S4A,B). As a result, the cytoplasmic β-catenin destruction complex cannot be formed and β-catenin is stabilized. In 2004, Yang et al. showed that LPAR2 and LPAR3 bind to the Gαq and that LPA increased the phosphorylation of GSK-3β and the subsequent activation of the β-catenin signaling pathway in the HCT116 cell line through PKC3β1 [37]. Therefore, there is a possibility that LPAR2 increases the phosphorylation of GSK-3β by activating the protein kinase C, which means that further studies are required to elucidate the mechanism responsible for the LPA-LPAR2-induced phosphorylation of GSK-3β and its downstream β-catenin signaling pathway in gastric cancer.

Cyclin D1 and c-MYC are well-known target genes of the β-catenin signaling pathway and are involved in oncogenic transformation [37]. Similarly, matrix metalloproteinase 2 (MMP-2) has been shown to play a vital role in tumor progression by regulating the tumor microenvironment, primarily by maintaining the connection between the tumor and stroma [38]. We observed profound increases in the expression of c-MYC, Cyclin D1, and MMP2 and the reversal of the LPA-induced effect after the addition of an LPAR2 receptor antagonist. Indeed, our most exciting observation was the significant overexpression of β-catenin and its downstream target genes in the clinical cohort studies of 415 human gastric cancer tissue samples, which is consistent with our data. These results confirm that LPA mediates its action in the initiation and progression of gastric cancer through the β-catenin signaling pathway and the LPAR2 receptor.

The metabolic pathway used by cancer cells differs from that of normal cells. Normal cells use mitochondrial OXPHOS as a major source of energy, whereas the metabolic phenotype of each cancer is different due to its wide range of heterogeneity [39]. When cancer cells encounter unfavorable conditions, they change their metabolic phenotype to adapt themselves to the new microenvironment [40]. In the 1920s, the German scientist Otto Warburg established that cancer cells use aerobic glycolysis because mitochondrial OXPHOS is impaired [41,42,43]. Several factors are responsible for the induction of aerobic glycolysis, including the loss of tumor suppressor genes and the activation of oncogenes, the hypoxic microenvironment, the mutation of mitochondrial DNA, and the origin of the tissues [44]. Later, several other research groups demonstrated that mitochondrial OXPHOS is intact in many cancers [45,46]. It has also been reported that rather than the impairment of OXPHOS, the Warburg effect in cancer occurs due to increased glycolysis, which leads to the suppression of OXPHOS; when glycolysis is inhibited, OXPHOS is re-established. Although glycolysis is the major pathway of the energy metabolism in cancer, some cancers use OXPHOS or a mixture of OXPHOS and glycolysis [47,48]. Our findings suggest that gastric cancer cells exposed to LPA experience increased mitochondrial bioenergetics by increasing OXPHOS and glycolysis. It has been shown that during the early stage of carcinogenesis, cancer cells use glycolysis to fulfill the energy demands due to the oncogenic transformation and hypoxic conditions. However, when the cells undergo aglycemic conditions and a shortage of nutrients due to an increased proliferation and migration of signaling proteins into the nucleus, the cells use OXPHOS as a source of energy [48]. Previous reports have suggested that cancer cells use both OXPHOS and glycolysis because when cancer cells grow rapidly, they need more energy and metabolic intermediates for the biosynthesis of macromolecules. Many intermediates of glycolysis and a truncated TCA cycle can be used to synthesize the macromolecules required for the rapid growth and proliferation of cancer cells [49]. In 2009, Dang et al. showed that the ectopic expression of MYC in cancers can drive aerobic glycolysis and/or OXPHOS depending on the tumor cell microenvironment. MYC increases glycolysis by increasing the glycolytic genes; however, it can increase OXPHOS through the activation of O2-dependent glutaminolysis which results from increases in glutaminase due to the reduced expression of miR23a/b [50]. In addition, as a result of glycolysis, more lactate is produced; this generates an acidic tumor microenvironment that helps in the invasion and metastasis of cancer cells [51]. Again, lactic acidosis favors OXPHOS by inhibiting glycolysis [40]. Since the results from our functional and biochemical experiments showed that LPA increases proliferation and the subsequent progression of a gastric cancer cell line, and that MYC expression is increased after an LPA treatment, there is a possibility that the LPA increased glycolysis and OXPHOS due to the increased MYC expression. Shin et al. showed that Axin is present in the mitochondria of HeLa cells; when it is present in the mitochondria, it causes instability of the mitochondrial complex IV, resulting in the reduction in OXPHOS and ATP synthesis [52]. In addition, Park and colleagues also demonstrated a reduced Axin1 expression in an E-cadherin-overexpressed AGS cell line that underwent EMT and increased OXPHOS as a source of energy [53]. Our study also found that an LPA treatment reduced the mRNA level of Axin2 which could be another possibility for the increased mitochondrial energy metabolism. Our results also suggested that LPA-induced OXPHOS and the aerobic glycolysis of cells were significantly reduced following a treatment with the LPAR2 antagonist and XAV393 (Axin stabilizer). These results confirmed that LPA mediates its action through the LPAR2 receptor during the malignant progression of gastric cancer cells by increasing the energy metabolism due to the dysregulation of Axin2. However, how LPA-LPAR2 and dysregulated Axin2 induce glycolysis and OXPHOS in the gastric cancer cell line was not clearly shown in this study, suggesting that a further investigation is required to reveal a more detailed molecular mechanism.

Taken together, all of our results demonstrate a novel role for LPA via its LPAR2 receptor in the malignant progression of gastric cancer by activating the β-catenin signaling pathway and by the alteration of the energy metabolism. We demonstrated that LPA-LPAR2 increased ATP production by both OXPHOS and glycolysis via the downregulation of Axin2, thereby providing energy sources for the proliferation, migration, and invasion of gastric cancer cells. Thus, targeting the specific LPAR2 receptor and Axin2 may provide a novel therapeutic approach for the treatment of gastric cancer.

## Data Availability

Not applicable.

## Supplementary Materials

The following supporting information can be downloaded at: https://www.mdpi.com/article/10.3390/biom12121805/s1, Figure S1: (**A**) Analysis of the mRNA levels of different LPA receptors in normal stomach and stomach cancer tissue using the TCGA dataset. LPAR2 levels were significantly increased in the stomach tumor samples compared to those of the normal stomach. (**B**) Analysis of the mRNA levels of the LPAR2 receptor in the different stages of stomach cancer using the TCGA dataset. LPAR2 levels were significantly increased in all stages of gastric cancer samples compared to those in normal stomach tissue. (**C**) LPAR2 is highly expressed in the AGS and NCI-N87 gastric cancer cell line compared to expression in the GES (normal gastric epithelial cell line) cell line. (**D**) Upper panel: Western blotting analysis of LPAR2 in the AGS, NCI-N87, and GES cell lines. Lower panel: quantification of the Western blotting results. (**E**) mRNA level of β-catenin was significantly increased in the AGS cell line. (**F**) β-catenin levels were significantly increased in AGS cell line after LPA treatment. All values are mean ± SD (*n* = 3). * *p* < 0.05, ** *p* < 0.01 compared to the groups. Figure S2: (**A**) Optimization of the LPA concentration used in the AGS cell line. Left panel: black line, control; red line, 5 µM LPA; green line, 10 µM LPA; yellow line, 20 µM LPA; right panel: quantification of the ECIS proliferation assay. (**B**) Quantification of the ECIS proliferation assay in the AGS cell line (**left panel**) and NCI-N87 cell line (**right panel**). (**C**) Quantification of the ECIS migration assay in AGS cell line (**left panel**) and NCI-N87 cell line (**right panel**). (**D**) scratch assay analysis of the effect of Ki16425 and the LPAR2 antagonist on LPA-induced migration in gastric cancer cells. The representative image was taken just after scratch and after 24 h of LPA, LPA + Ki16425 or LPA+LPAR2 antagonist treatment in AGS (**upper**) or NCI-N87 (**lower**) (scale bars, 100 µm). (**E**) LPA receptor antagonists reduced LPA-induced invasion activity in gastric cancer cells. Representative images of invaded cells obtained from the transwell invasion assay. **Left**: AGS cell; **right**; NCI-N87 cell. All values are mean ± SD (*n* = 3). * *p* < 0.05, ** *p* < 0.01 compared to the control. The Student’s *t*-test was used for comparisons between two groups; one-way ANOVA followed by the Bonferroni post hoc test was performed for more than two groups. Figure S3: (**A**) LPA induced migration activity was reduced in LPAR2 knockdown AGS (**Upper panels**) or NCI-N87 (**lower panels**) cells. The representative image was taken just after scratch and after 24 h of LPA treatment. (**B**) Invasion assay in AGS cell line (**left panel**) or the NCI-N87 cell line (**right panel**) following transduction of the cells by a vector or an LPAR2 sh-RNA. All values are mean ± SD (*n* = 3). ** *p* < 0.01; one-way ANOVA) followed by the Bonferroni post hoc test. Figure S4: (**A**) LPA treatment decreased the mRNA level of Axin2 in the AGS (**left**) and NCI-N87 (**right**) cell lines. (**B**) Western blotting analysis of AGS (**upper**) and NCI-N87 (**lower**) cell lines to investigate LPA treatment-induced phosphorylation of P-GSK-3β. Respective lower bar graph in each panel: quantification of P-GSK-3β normalized to total- GSK-3β. All values are mean ± SD (*n* = 3). ** *p* < 0.01; one-way ANOVA) followed by the Bonferroni post hoc test. Figure S5: Upper panels: Western blotting analysis of LPAR2 knockdown in the AGS (**left**) and NCI-N87 (**right**) cell line using three different shRNA (shLPAR21-3). Lower panels: quantification of western blotting results. All values are mean ± SD (*n* = 3). ** *p* < 0.01; one-way ANOVA) followed by the Bonferroni post hoc test. Figure S6: (**A**) Quantification of the ECIS proliferation assay in AGS cell line after treating the cells with LPA or LPA with XAV393. (**B**) Representitive image of the scratch assay in AGS cell line after treating the cells with LPA or LPA with XAV393. (**C**) Representitive image of the invasion assay in AGS cell line after treating the cells with LPA or LPA with XAV393. All values are mean ± SD (*n* = 3). ** *p* < 0.01; one-way ANOVA) followed by the Bonferroni post hoc test. Figure S7: Analysis of mRNA level of β-catenin (CTNNB1) and its downstream target genes in the normal stomach and stomach cancer using the TCGA dataset. * *p* < 0.05.
