# Supplementary material for: Alteration of Cellular Energy Metabolism through LPAR2-Axin2 Axis in Gastric Cancer
Source: Biomolecules. 2022 Dec 2;12(12):1805. doi: 10.3390/biom12121805 (PMC9775664; doi:10.3390/biom12121805)
Supplement: Supplementary file 1 [file biomolecules-12-01805-s001.zip › biomolecules-2008608-supplementary.pdf]

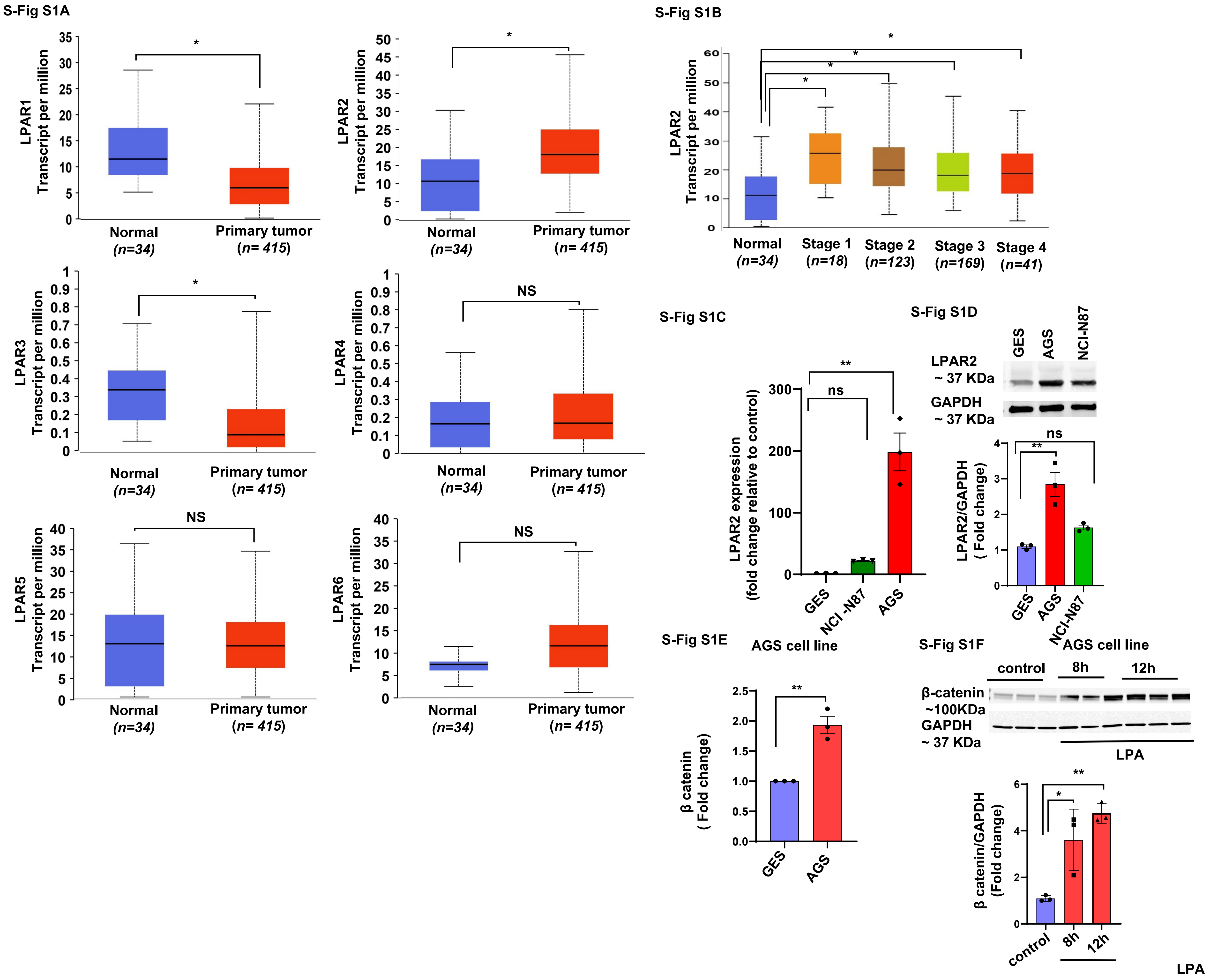

Supplementary Figure S1.A). Analysis of the mRNA levels of different LPA receptors in normal stomach and stomach cancer tissue using the TCGA dataset. LPAR2 levels were significantly increased in the stomach tumor samples compared to those of the normal stomach. B). Analysis of the mRNA levels of the LPAR2 receptor in the different stages of stomach cancer using the TCGA dataset. LPAR2 levels were significantly increased in all stages of gastric cancer samples compared to those in normal stomach tissue. C). LPAR2 is highly expressed in the AGS and NCI-N87 gastric cancer cell line compared to expression in the GES (normal gastric epithelial cell line) cell line. D) Upper panel: Western blotting analysis of LPAR2 in the AGS, NCI-N87, and GES cell lines. Lower panel: quantification of the Western blotting results. E) mRNA level of β-catenin was significantly increased in the AGS cell line. F) β-catenin levels were significantly increased in AGS cell line after LPA treatment. All values are mean ± SD (n=3).* P<0.05, ** P<0.01 compared to the control. The Student's t-test was used for comparisons between two groups.

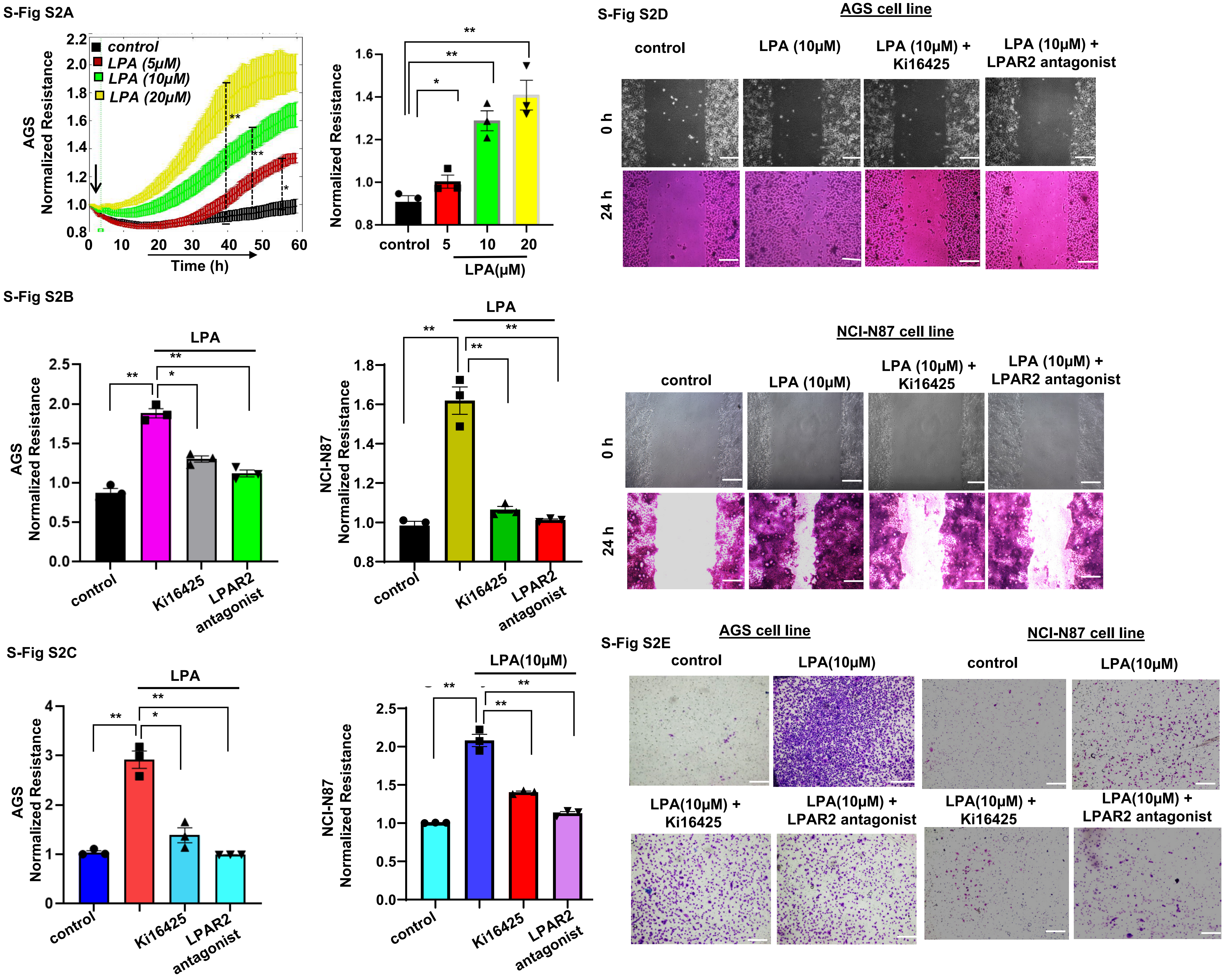

Supplementary Figure S2 .A) Optimization of the LPA concentration used in the AGS cell line. Left panel: black line, control; red line, 5 μM LPA; green line, 10 μM LPA; yellow line, 20 μM LPA; right panel: quantification of the ECIS proliferation assay.B) Quantification of the ECIS proliferation assay in the AGS cell line(left panel) and NCI-N87 cell line (right panel). C) Quantification of the ECIS migration assay in AGS cell line (left panel) and NCI-N87 cell line (right panel). D) scratch assay analysis of the effect of Ki16425 and the LPAR2 antagonist on LPA-induced migration in gastric cancer cells. The representative image was taken just after scratch and after 24 h of LPA, LPA + Ki16425 or LPA+LPAR2 antagonist treatment in AGS (upper) or NCI-N87 (lower) (scale bars, 100 μm). E) LPA receptor antagonists reduced LPA-induced invasion activity in gastric cancer cells. Representative images of invaded cells obtained from the transwell invasion assay. Left : AGS cell; right; NCI-N87 cell. All values are mean ± SD (n=3).* P<0.05, ** P<0.01 compared to the control. The Student's t-test was used for comparisons between two groups; one-way ANOVA followed by the Bonferroni post hoc test was performed for more than two groups.

.

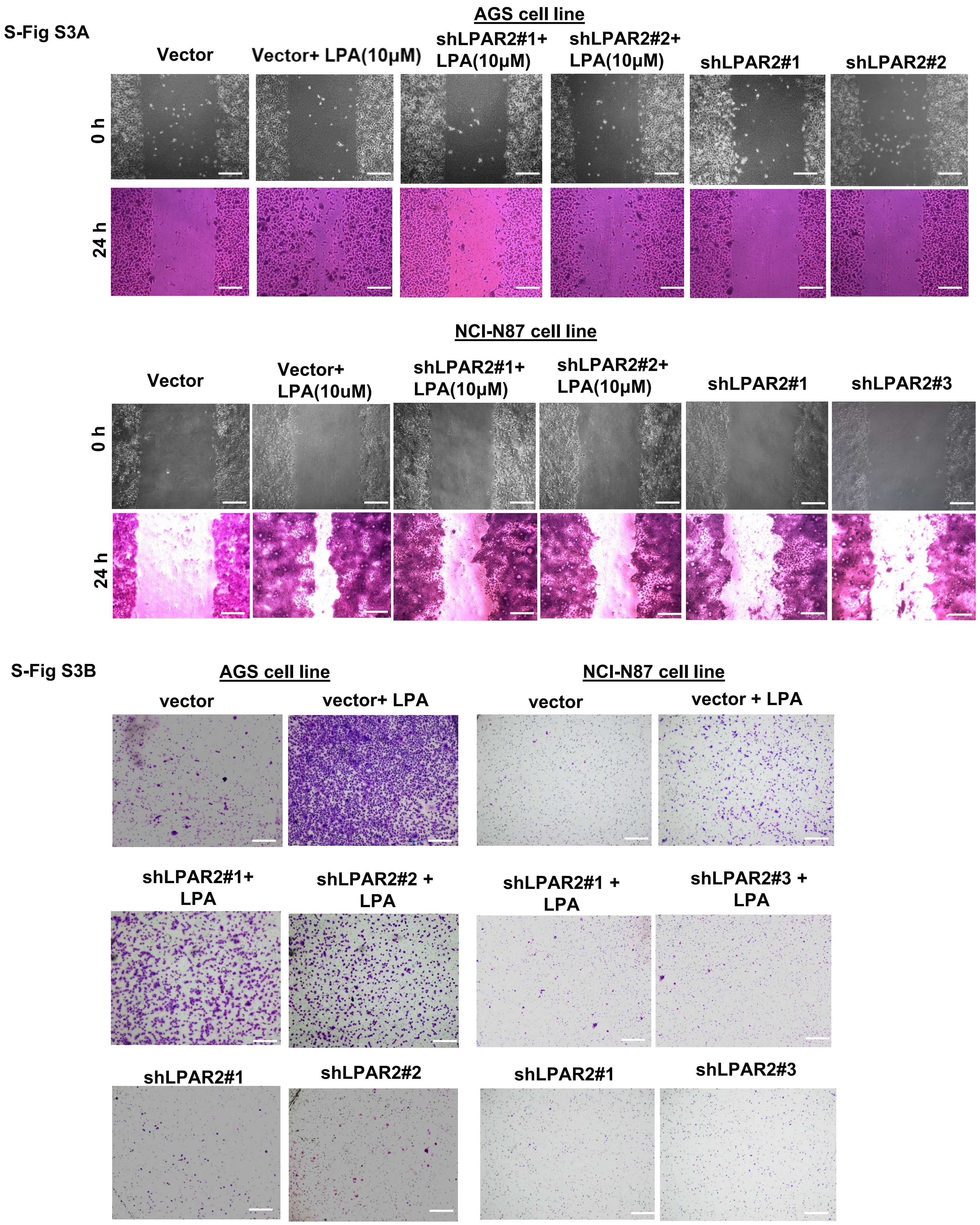

Supplementary Figure S3. A) LPA induced migration activity was reduced in LPAR2 knockdown AGS (Upper panels) or NCI-N87 (lower panels) cells. The representative image was taken just after scratch and after 24 h of LPA treatment. B) Invasion assay in AGS cell line(left panel) or the NCI-N87 cell line(right panel) following transduction of the cells by a vector or an LPAR2 sh-RNA. All values are mean ± SD (n=3). ** P<0.01; one-way ANOVA) followed by the Bonferroni post hoc test.

S-Fig S4A

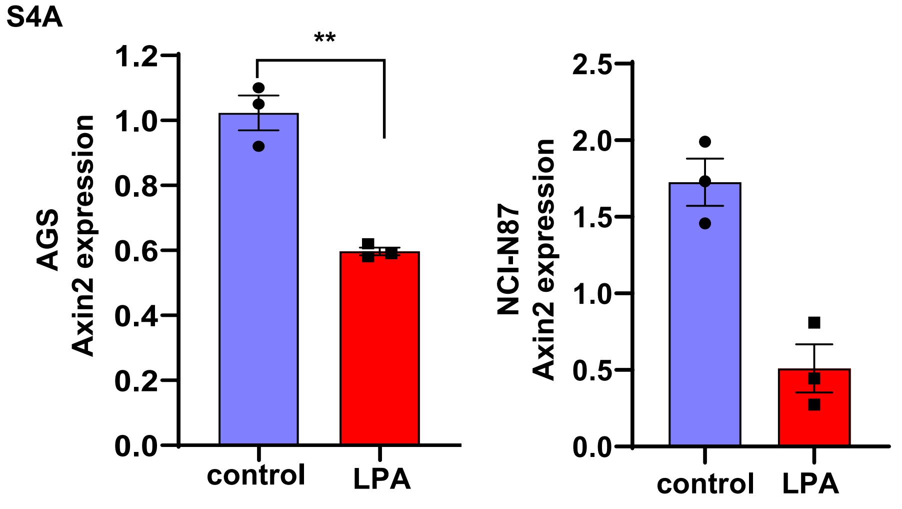

S-Fig S4B

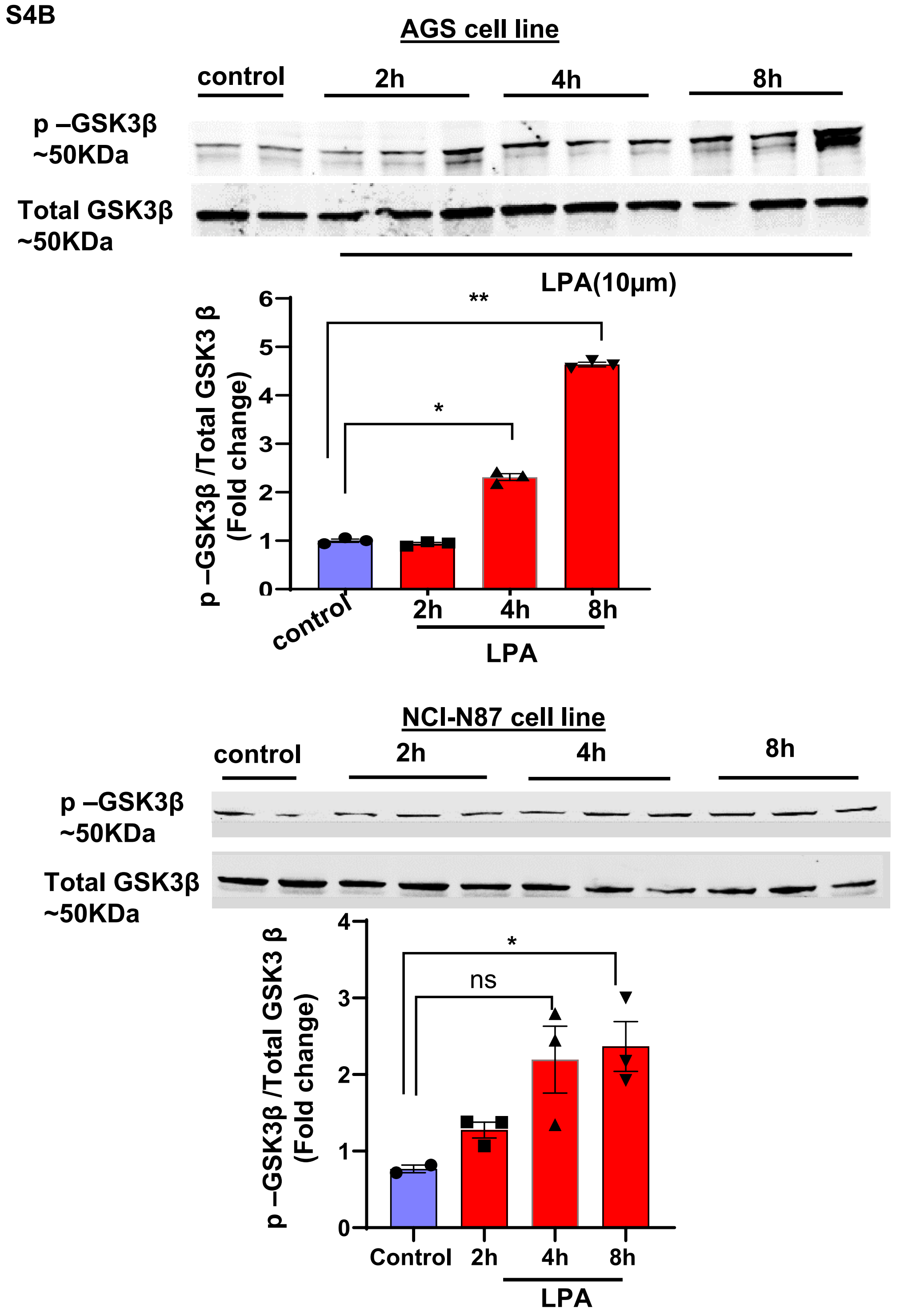

Supplementary Figure S4. A) LPA treatment decreased the mRNA level of Axin2 in the AGS (left) and NCI-N87(right) cell lines. B) Western blotting analysis of AGS(upper) and NCI-N87 (lower) cell lines to investigate LPA treatment-induced phosphorylation of P-GSK-3β. Respective lower bar graph in each panel: quantification of P-GSK-3β normalized to total- GSK-3β. All values are mean ± SD (n=3). ** P<0.01; one-way ANOVA) followed by the Bonferroni post hoc test

S-Fig S5

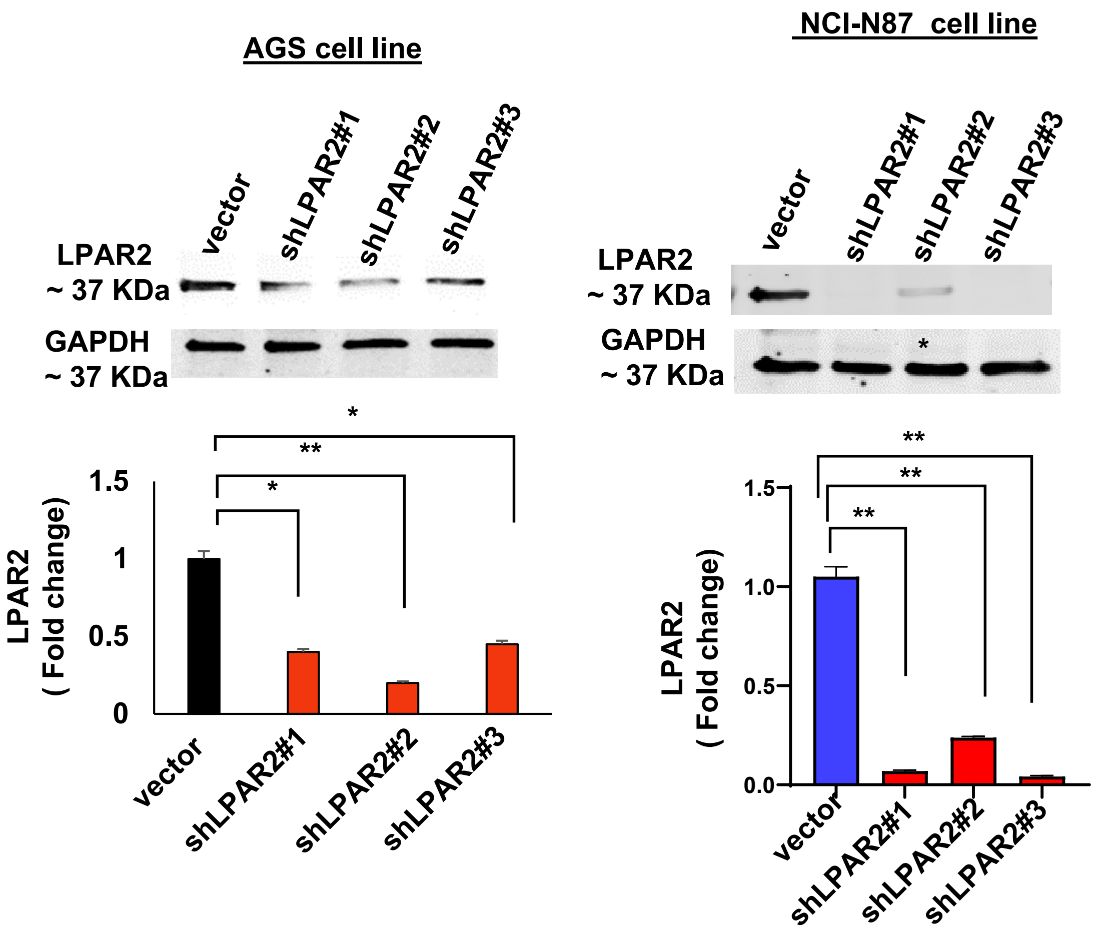

Supplementary Figure S5. Upper panels: Western blotting analysis of LPAR2 knockdown in the AGS(left) and NCI-N87(right) cell line using three different shRNA (shLPAR21-3).Lower panels: quantification of western blotting results. All values are mean ± SD (n=3). ** P<0.01; one-way ANOVA) followed by the Bonferroni post hoc test.

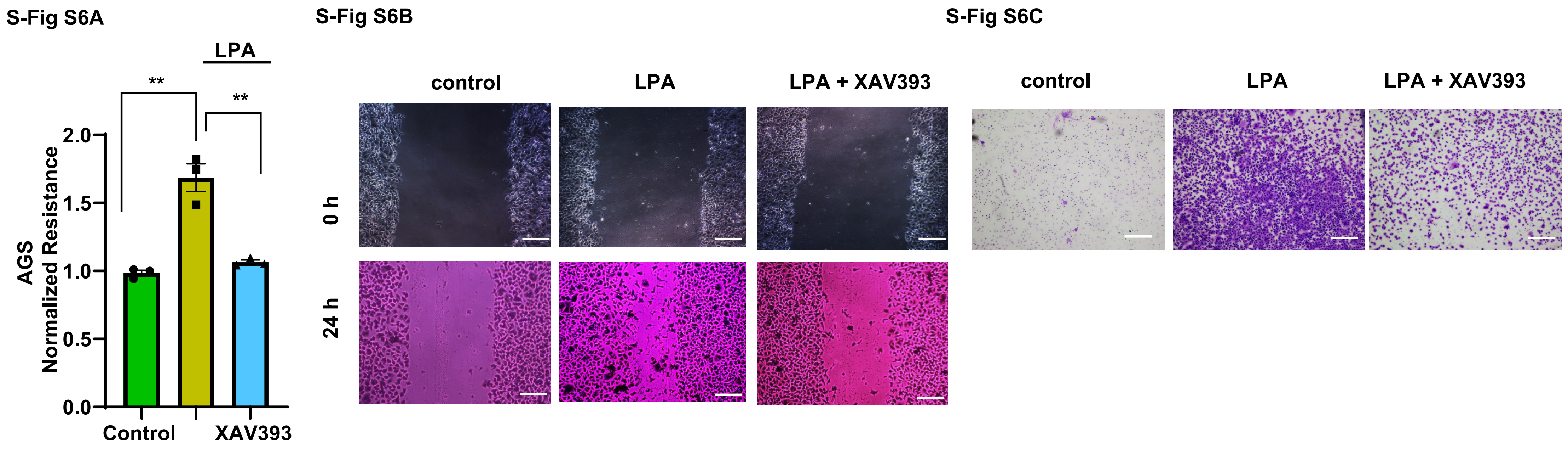

Supplementary Figure S6.A) Quantification of the ECIS proliferation assay in AGS cell line after treating the cells with LPA or LPA with XAV393. B) Representitive image of the scratch assay in AGS cell line after treating the cells with LPA or LPA with XAV393. C) Representitive image of the invasion assay in AGS cell line after treating the cells with LPA or LPA with XAV393. All values are mean ± SD (n=3). ** P<0.01; one-way ANOVA) followed by the Bonferroni post hoc test.

S-Fig S7

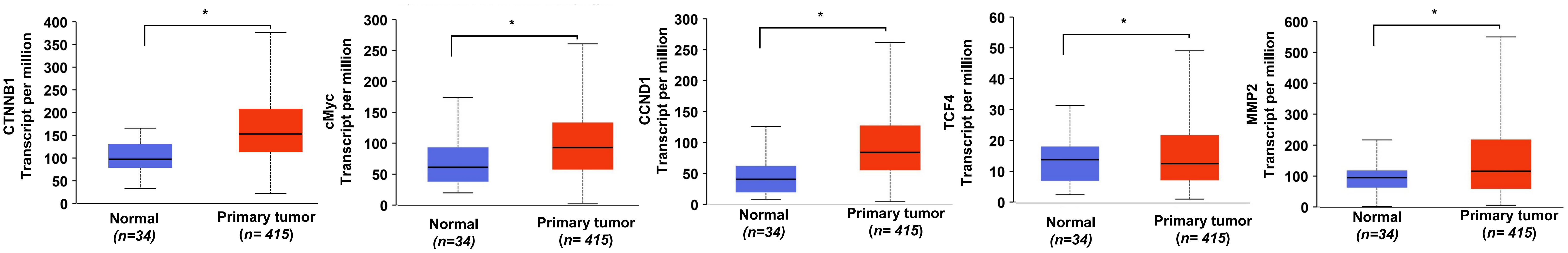

Supplementary Figure S7. Analysis of mRNA level of β-catenin (CTNNB1) and its downstream target genes in the normal stomach and stomach cancer using the TCGA dataset. * P<0.05.
